# Tenofovir disoproxil fumarate may not cause renal and bone toxicity in chronic hepatitis B patients: a retrospective cross-sectional study

**DOI:** 10.3906/sag-1805-233

**Published:** 2019-02-11

**Authors:** Rahmet GÜNER, Zeliha KOÇAK TUFAN, Gül Ruhsar YILMAZ, Mehmet A. TAŞYARAN

**Affiliations:** 1 Department of Infectious Diseases and Clinical Microbiology, Ankara Atatürk Training and Research Hospital,Yıldırım Beyazıt University, Ankara Turkey2; 2 Department of Infectious Diseases and Clinical Microbiology, Faculty of Medicine, Süleyman Demirel University, Isparta Turkey

**To the Editor,**

Tenofovir (TDF) is a nucleotide reverse transcriptase inhibitor used for treatment of chronic hepatitis B (CHB). TDF-associated hypophosphatemia has been reported in the literature [1,2]. However, randomized double-blind placebo-controlled trials have documented a favorable renal safety profile of TDF in patients with normal baseline renal function and a comparable incidence of hypophosphatemia [3]. 

In this retrospective cross-sectional study, we aimed to investigate if there was a significant change in PO4, Ca levels, glomerular filtration rate (GFR), and bone mineral densitometry (BMD) of CHB patients who were under TDF therapy. Fifty-seven adult CHB patients treated with TDF between 2008 and 2013 were included. Local Ethical Committee approval of the hospital was obtained. Analyses were performed by using SPSS for Windows version 12 (SPSS Inc, Chicago, IL).

Median TDF therapy duration was 35 (6–68) months. Although only 3 had hypophosphatemia (<2.5 mg/dL), 15 patients had relatively low phosphate levels (<3 mg/dL) at baseline (a normal adult is expected to have a PO4 level of 3–4.5 mg/dL). Seventeen (29.8%) of all patients had experienced PO4 levels below 2.5 mg/dL during the therapy period once or several times. Apart from three patients, all (82%) of them had mild hypophosphatemia (2–2.5 mg/dL). The critical values for PO4 and Ca were not detected in any of the patients. Hypocalcemia and hypophosphatemia were present in 2 (3.8%) patients at 45 and 68 months of therapy. No correlation was found between the Ca and PO4 levels and age, sex, or duration of the therapy. The mean urea, creatinine levels, and GFRs at baseline and last visit are given in Table. 

**Table 1:** The mean urea, creatinine levels, and GFRs at baseline and at last visit.

	Baseline	Last visit	P
Urea (mg/dL)	29.5 ± 8.3	29.6 ± 9.4	NS
Creatinine (mg/dL)	0.9 ± 0.3	0.9 ± 0.2	NS
Median GFR (min–max)	92 (39–169)	104 (47–181)	NS
PO_4_	3.2 ± 0.7	3.1 ± 0.5	NS
Ca	9.3 ± 0.4	9.5 ± 0.4	NS

Values are presented as mean ± standard deviation unless otherwise indicated. NS; not significant. Baseline: At the beginning of the therapy. Last visit: The last visit for each patient who are under treatment.

None of the patients had a sharp decrease in GFR (<30 mL/min/1.73 m2). Three patients (5.3%) had a GFR level of 30–59 mL/min/1.73 m2 both at baseline and at the end of treatment. BMD results of 31 patients (75.6%) were normal while 8 (19.5%) had osteopenia and 2 (4.8%) had osteoporosis. 

Renal and bone toxicity and PO4 and Ca levels of CHB patients under TDF therapy were evaluated in this study. None of the patients experienced severe hypophosphatemia and no significant change was observed in mean urea, creatinine, and GFR levels.

Although hypophosphatemia was defined as below 2.5 mg/dL, a normal adult is expected to have a PO4 level of 3–4.5 mg/dL and interestingly 40% of our patients already had a PO4 level below 3 mg/dL at baseline in our study. Preexisting risk factors for renal disease, such as diabetes, hypertension, coinfection with HIV or HCV, and baseline renal impairment, coadministration of nephrotoxic medications, and increasing age, can be reasons for hypophosphatemia [4]. Though not persisted, 30% of our study group showed a mild hypophosphatemia during the study period. Interestingly, there was no correlation between the minimum PO4 levels and duration of the therapy. If we consider the duration of the treatment, half of our patients were under long term therapy of more than 36 months and only two patients had hypophosphatemia which was mild in intensity. In a large phase-3 study, hypophosphatemia was reported as 1.5% and increase in creatinine by 0.5 mg/dL was 1.7% in the 7th year of TDF treatment of CHB patients [5]. Renal functions of our patients did not differ at a significant level compared with baseline. None of the patients had renal failure nor showed a third stage decrease in GFR. In another study evaluating TDF versus emtricitabine + TDF, no significant difference regarding renal functions and no increased risk for hypophosphatemia was reported [6]. 

In conclusion, TDF did not seem to alter PO4 and Ca levels, GFR, and BMD at a significant level in a real‑life setting, and close monitoring is recommended to minimize the risk.

## References

[ref0] (2014). Bone scintigraphy and secondary osteomalacia due to nephrotoxicity in a chronic hepatitis B patient treated with tenofovir. Rev Esp Med Nucl Imagen Mol.

[ref1] (2016). Hypophosphatemic osteomalacia induced by tenofovir in HIV-infected patients. Clin Rheumatol.

[ref2] (2006). Evaluation of hypophosphatemia in tenofovir disoproxil fumarate (TDF)-exposed and TDF-unexposed HIV-infected out-patients receiving highly active antiretroviral therapy. HIV Medicine.

[ref3] (2013). Hypophosphataemia with non-tenofovir-containing antiretroviral therapy. Int J STD AIDS.

[ref4] (2015). Dig Dis Sci.

